# Supplementary material for: Improved electrochemical performance of multi-walled carbon nanotube reinforced gelatin biopolymer for transient energy storage applications
Source: PLoS One. 2023 Nov 9;18(11):e0288113. doi: 10.1371/journal.pone.0288113 (PMC10635558; doi:10.1371/journal.pone.0288113)
Supplement: S1 Table — (DOCX) [file pone.0288113.s001.docx]

**Improved electrochemical performance of Multi-walled carbon nanotube reinforced Gelatin biopolymer for transient energy storage applications**

Rabeya Binta Alam, Md. Hasive Ahmad, Muhammad Rakibul Islam*

Department of Physics, Bangladesh University of Engineering and Technology (BUET), Dhaka, Bangladesh

*Corresponding Author

E-mail:rakibul@phy.buet.ac.bd

**Table ST 1. Optimization Table**

| **Parameters** | **Optimized Quantity** |
| --- | --- |
| Polymer Matrix (Gelatin) | 5 g |
| Solvent (DI water) | 25 mL |
| Plasticizer (Glycerol) | 2 mL |
| Stirring Temperature | 70 ⁰C |
| Stirring time | 30 minutes |
| Drying Temperature | 40 ⁰C |
