# Supplementary material for: Improved electrochemical performance of multi-walled carbon nanotube reinforced gelatin biopolymer for transient energy storage applications
Source: PLoS One. 2023 Nov 9;18(11):e0288113. doi: 10.1371/journal.pone.0288113 (PMC10635558; doi:10.1371/journal.pone.0288113)
Supplement: Attachment — Submitted filename: Referee Comments.doc [file pone.0288113.s002.doc]

**Manuscript Number: PONE-D-23-03580**

**Comments**

1. Title of the manuscript should be modified. It should be precise as well as catchy.

2. The optimization study for the experimental section should be added as supplementary information.

3. The steps for electrode preparation must be included in the manuscript. Also, check the working electrode that authors are using in the electrode set up. Also, check the mass of the electrode before and after the deposition of the material.

3. In figure 1, why is dip for the peak in the wavenumber 2000-3000 cm^-1^ (corresponding to Gel/0.01% MWCNT) not prominent?

4 The trends for the change in the morphology from Fig 2 (d-f) need explanation.

5 Mention the frequency range and voltage employed for EIS data.

6. Authors have already published one manuscript with doi.org/10.1016/j.heliyon.2021.e07468. The results and scientific explanation of the present manuscript almost resemble with the published manuscript. I did not find any novelty in the present manuscript. What is the significance of MWCNT in comparison to SWCNT? Authors should highlight all important aspect of the present work. Moreover, the analysis of the obtained experimental results need modification.
