# Supplementary material for: Improved electrochemical performance of multi-walled carbon nanotube reinforced gelatin biopolymer for transient energy storage applications
Source: PLoS One. 2023 Nov 9;18(11):e0288113. doi: 10.1371/journal.pone.0288113 (PMC10635558; doi:10.1371/journal.pone.0288113)
Supplement: Attachment — Submitted filename: POne_Reviewers _ response_R1.docx [file pone.0288113.s003.docx]

**Reply to the Referees Comments on Manuscript Number: PONE-D-23-03580**

We thank the referees for the critical review of the manuscript. In the following, we provide our responses to the referee’s comments.

**Reply to the First referee’s comments:**

**General Comments:** Dear Authors After carefully reading this manuscript, I would like to recommend for publishing after miner revision
**Response:** We thank the referee for his/her thoughtful comment.

**Comment (1)**   The English need to rechecking to avoid some mistake in Abstract and introduction.

**Response 1:** We thank the referee for his/her comments. The grammatical mistakes and typos throughout the manuscript have corrected. Please see the list of changes #1.

**Comment (2)**   Update the same references in the Introduction.

**Response 2:** We thank the referee for the comment. Following the referee’s suggestion several updated references have been included in the introduction part of the revised manuscript. Please see the list of changes # 2.

**Reply to the Second referee’s comments:**

**Comment (1)**     Title of the manuscript should be modified. It should be precise as well as catchy.

**Response 1:** We thank the referee for the comment. Following the referee’s suggestion we change the title of the article to make it precise and catchy. Please see the list of changes # 3.

**Comment (2)**     The optimization study for the experimental section should be added as supplementary information.

**Response 2:** We thank the referee for the comment. While preparing the nanocomposites we have taken several concentrations of MWCNT nanofiller and studied their effect on the electrochemical performance of the nanocomposites. For the preparation of the nanocomposite, we followed the procedure published in one of our previous work at: doi.org/10.1016/j.heliyon.2021.e07468.

**Comment (3)** The steps for electrode preparation must be included in the manuscript. Also, check the working electrode that authors are using in the electrode set up. Also, check the mass of the electrode before and after the deposition of the material.

**Response 3:** We thank the referee for the comment. Following the referee’s suggestion the electrode preparation steps are included in the revised version of the manuscript. Please see the list of changes #4.

**Comment (4)**     In figure 1, why is dip for the peak in the wavenumber 2000-3000 cm-1 (corresponding to Gel/0.01% MWCNT) not prominent?

**Response 4:** We thank the referee for the comment. In the FTIR spectra of Figure 1, the dip between 2000-3000 cm^-1^ occurred due to the presence of CO_2_ present in the environment.

**Comment (5)**     The trends for the change in the morphology from Fig 2 (d-f) need explanation.

**Response 5:** We thank the referee for the comment. Figure 2 (d-f) represents the surface morphology of Gel/MWCNT composites at higher concentrations of MWCNT. With the increase of the concentration of MWCNT the roughness of the surface got increased and lots of fibrous pattern are observable on the surface. The Gel matrix might wrapped the MWCNT and create fibrous pattern. This discussion has been included in the revised version of the manuscript. Please see the list of changes #5.

**Comment (6)**     Mention the frequency range and voltage employed for EIS data.

**Response 6:** We thank the referee for the comment. The EIS data was taken in the frequency range between 10^-1^ Hz to 10^6^ Hz and at an AC voltage of 10 mV. This information has been included in the revised manuscript. Please see the list of changes #6.

**Comment (7)** Authors have already published one manuscript with doi.org/10.1016/j.heliyon.2021.e07468. The results and scientific explanation of the present manuscript almost resemble with the published manuscript. I did not find any novelty in the present manuscript. What is the significance of MWCNT in comparison to SWCNT? Authors should highlight all important aspect of the present work. Moreover, the analysis of the obtained experimental results need modification.

**Response 7:** We thank the referee for the comment. It is right our group has published a paper focusing on the effect of SWCNT on the dielectric performance of Gelatin biopolymer. However, the effect of MWCNT on such biopolymers has never been studied in detail. Even though both SWCNT and MWCNT are carbon nanotubes but they possess significant differences: in terms of physical properties, cost efficiency, and application point of view. The MWCNTs are consisting of cylindrical tubes that are larger compared to the SWCNTs and are mechanically stronger. Additionally, SWCNTs are a mixing of tubes that are both semiconducting and metallic in nature whereas the MWCNTs are mostly metallic. These physical aspects of the nanofiller can help to affect the physical properties of the polymer matrix. Also, the MWCNTs are much cheaper compared to SWCNTs. In the paper (doi.org/10.1016/j.heliyon.2021.e07468) we found that the incorporation of SWCNT can increase the specific capacitance of the nanocomposite by about four times from 124 mF/g to 467 mF/g. In this article, we see that the incorporation of MWCNT can increase the specific capacitance of the nanocomposite by 100 times from 0.12 F/g to 12.7 F/g. Following the referee’s suggestion these discussions are included in the revised manuscript. Please see the list of changes no. #7.

**Additional Comment:** The authors should include few recent references like; Thermoelectric-powered supercapacitor based on Ni-Mn nanowires driven by quadripartite electrolyte; Role of Electrochemical techniques for photovoltaic and supercapacitor applications; etc.

**Response:** We thank the referee for the comment. Following the referee’s suggestion several updated and relevant references have been incorporated in the revised manuscript. Please see the list of changes #2.

**List of Changes on Manuscript ID: PONE-D-23-03580**

1. The grammatical mistakes and typos throughout the manuscript has corrected.
2. A number of references e.g. [2], [4], [10] and [11] has been replaced by updated and relevant references included in the revised manuscript
3. The title of the reference has been changed from “Multi-walled carbon nanotube reinforced Gelatin biopolymer with improved electrochemical performance and cyclic stability for transient energy storage applications” to “*Improved electrochemical performance of Multi-walled carbon nanotube reinforced Gelatin biopolymer for transient energy storage applications*”
4. In the Characterization techniques section of the revised manuscript the following sentences “*The working electrodes were prepared by depositing the slurry of active material. To prepare the slurry the active materials were mixed with Polyvinyl alcohol (PVAand Dimethyl sulfoxide. Then the rsulting mixtures were then sonicated for few hours followed by deposition on a glassy carbon electrode. Then the working electrodes were dried for several hours at 70 ⁰C. The mass of the active materials were found almost same before and after electrochemical measurements*.” has been included
5. In the Surface morphology section of the revised manuscript the sentences “*With the increase of the concentration of MWCNT the roughness of the surface got increased and lots of fibrous pattern are observable on the surface. The Gel matrix might wrapped the MWCNT and create fibrous pattern.*” Has been included.
6. In the Electrochemical impedance spectroscopy part of the revised manuscript the sentences “The electrochemical impedance spectroscopy (EIS) was performed to analyze the electrochemical behavior of charge and ion transportation between the electrolyte and the surface of the material and is shown in Fig. 8.” has changed to “*The electrochemical impedance spectroscopy (EIS) was performed to analyze the electrochemical behavior of charge and ion transportation between the electrolyte and the surface of the material. The EIS data was taken in the frequency range between 10^-1^ Hz to 10^6^ Hz and at an AC voltage of 10 mV and is shown in Fig. 8.*”
7. In the Electrochemical Properties section of the revised manuscript the sentences “*Recently, Rabeya et. al. [37] synthesized SWCNT/Gel nanocomposite and showed the incorporation of SWCNT increase the specific capacitance from 124 mF/g to 467 mF/g. The MWCNTs can therefore be considered as the suitable choice as the nanofiller over SWCNTs for energy storage applications*.” has been included.
