# Supplementary material for: Improved electrochemical performance of multi-walled carbon nanotube reinforced gelatin biopolymer for transient energy storage applications
Source: PLoS One. 2023 Nov 9;18(11):e0288113. doi: 10.1371/journal.pone.0288113 (PMC10635558; doi:10.1371/journal.pone.0288113)
Supplement: Attachment — Submitted filename: Referee Comments.doc [file pone.0288113.s004.doc]

**Comment:**

All the comments are well addressed excluding one. An optimisation table for the experimental part should be provided, and the rationale for selecting the particular concentration in the synthesis process should be clarified.
