# Supplementary material for: Improved electrochemical performance of multi-walled carbon nanotube reinforced gelatin biopolymer for transient energy storage applications
Source: PLoS One. 2023 Nov 9;18(11):e0288113. doi: 10.1371/journal.pone.0288113 (PMC10635558; doi:10.1371/journal.pone.0288113)
Supplement: Attachment — Submitted filename: POne_Reviewers _ response_R2.docx [file pone.0288113.s005.docx]

**Reply to the Referees Comments on Manuscript Number: PONE-D-23-03580R1**

We thank the referees for the critical review of the manuscript. In the following, we provide our responses to the referee’s comments.

**Reply to the second referee’s comments:**

**Comment:** All the comments are well addressed excluding one. An optimisation table for the experimental part should be provided, and the rationale for selecting the particular concentration in the synthesis process should be clarified.
**Response:** We thank the referee for his/her thoughtful comment. Following referee’s comment an optimization table has been included in the revised version of the manuscript.

Here in this article we have studied the effect of concentration of multi walled carbon nanotube (MWCNT) nanofiller on the electrochemical performance of biodegradable polymer (gelatin). Here we have taken the concentration of MWCNT as 0.005, 0.01, 0.02, 0.05 wt. %.

For higher concentration of MWCNTs the nanotubes can agglomerate and which may results in a decrement of the electrochemical performance. That is why we kept the concentration of MWCNTs low.

**List of Changes on Manuscript ID: PONE-D-23-03580R1**

1. In the Synthesis of Gel/MWCNT nanocomposite sub section of the revised manuscript the following sentence “*For higher concentration of MWCNTs the nanotubes can agglomerate and which may results in a decrement of the electrochemical performance and that is why the concentration of nanofillers were kept low*.” has been included
2. In the Synthesis of Gel/MWCNT nanocomposite sub section of the revised manuscript the following sentences “*The table ST1 in the supplementary section shows the optimized parameters required for the synthesis of nanocomposite.*” has been included.
3. A supplementary section has been incorporated.
